# A New Methodology of Viewing Extra-Axial Fluid and Cortical Abnormalities in Children with Autism via Transcranial Ultrasonography

**DOI:** 10.3389/fnhum.2013.00934

**Published:** 2014-01-15

**Authors:** James Jeffrey Bradstreet, Stefania Pacini, Marco Ruggiero

**Affiliations:** ^1^Newport Brain Research Laboratory, Newport Beach, CA, USA; ^2^Brain Treatment Center, Newport Beach, CA, USA; ^3^Brain Treatment Center, Buford, GA, USA; ^4^Department of Experimental and Clinical Medicine, University of Florence, Florence, Italy; ^5^Department of Experimental and Clinical Biomedical Sciences, University of Florence, Florence, Italy; ^6^Immuno Biotech Ltd., Saint Peter Port, Guernsey

**Keywords:** transcranial ultrasound, autism spectrum disorders, extra-axial fluid, cortical dysplasia, cerebral spinal fluid

## Abstract

**Background:** Autism spectrum disorders (ASDs) are developmental conditions of uncertain etiology which have now affected more than 1% of the school-age population of children in many developed nations. Transcranial ultrasonography (TUS) via the temporal bone appeared to be a potential window of investigation to determine the presence of both cortical abnormalities and increased extra-axial fluid (EAF).

**Methods:** TUS was accomplished using a linear probe (10–5 MHz). Parents volunteered ASD subjects (*N* = 23; males 18, females 5) for evaluations (mean = 7.46 years ± 3.97 years), and 15 neurotypical siblings were also examined (mean = 7.15 years ± 4.49 years). Childhood Autism Rating Scale (CARS2^®^) scores were obtained and the ASD score mean was 48.08 + 6.79 (Severe).

**Results:** Comparisons of the extra-axial spaces indicated increases in the ASD subjects. For EAF we scored based on the gyral summit distances between the arachnoid membrane and the cortical pia layer (subarachnoid space): (1) <0.05 cm, (2) 0.05–0.07 cm, (3) 0.08–0.10 cm, (4) >0.10 cm. All of the neurotypical siblings scored 1, whereas the ASD mean score was 3.41 ± 0.67. We also defined cortical dysplasia as the following: hypoechoic lesions within the substance of the cortex, or disturbed layering within the gray matter. For cortical dysplasia we scored: (1) none observed, (2) rare hypoechogenic lesions and/or mildly atypical cortical layering patterns, (3) more common, but separated areas of cortical hypoechogenic lesions, (4) very common or confluent areas of cortical hypoechogenicity. Again all of the neurotypical siblings scored 1, while the ASD subjects’ mean score was 2.79 ± 0.93.

**Conclusion:** TUS may be a useful screening technique for children at potential risk of ASDs which, if confirmed with repeated studies and high resolution MRI, provides rapid, non-invasive qualification of EAF, and cortical lesions.

## Introduction

Autism and related spectrum disorders (ASDs) have become common childhood developmental disorders which greatly impact the individual’s quality of life. In a recent U.S. survey, the estimated prevalence of ASDs, averaged among the 14 special data capture sites, was 11.3 per 1,000 (1 in 88) children aged 8 years (Centers for Disease Control and Prevention, 2012). The prevalence varied widely across all sites (range: 4.8–21.2 per 1,000 children aged 8 years). While the prevalence data from different countries is difficult to compare due to data collection methodologies, it is generally accepted the U.S. data is reflective of trends observed in other developed nations. Given these prevalence data, more widely accessible methods of quantifying early central nervous system abnormalities are urgently needed.

A precise cause for the apparent pandemic occurrence of autism has yet to be defined (Chaste and Leboyer, 2012). However, recent studies point to a complex combination of immunological, genetic, epigenetic, infectious, and environmental factors which may contribute to the onset and development of the disorder (Gesundheit et al., 2013; Siniscalco et al., 2013). Pathophysiology and histopathological standards are not widely accepted, and observations based on formalin-fixed brains (brain-banked specimens) may lose features present in living tissues. Despite these limitations, various pathologies have been observed in the histology of ASDs. Of interest to our evaluations, cortical dysplasias, most of which could not be explained by either abnormal neurogenesis or defective neuronal migration, were reported in 13 age-matched autism cases (Wegiel et al., 2010). Within the case series, only 7 subjects were ages 21 or less, thus diminishing our confidence that these findings are of etiological, rather than of secondary significance. In that study, multifocal cerebral dysplasia with local distortion of the cytoarchitecture of the neocortex was noted in four brains (31%), and of the entorhinal cortex in two brains (15%). Nonetheless, some of the described anatomical locations of the dysplasias were in areas we felt could be observed with a novel technique of transcranial ultrasonography (TUS) which we will present in this study.

An alternate theory of autistic cortical dysfunction is that minicolumn organization is aberrant and thus disruptive to organized neurotransmission (Casanova et al., 2002). In that report, prefrontal and temporal lobe abnormalities were detected in the microstructure of neocortical minicolumns. While the cortical minicolumn is conserved in all mammals, humans are distinct in their greater width of and microstructure within the minicolumns (Raghanti et al., 2010). In a recent more detailed report (Casanova et al., 2010), this abnormality was reconfirmed. Casanova’s group observed neurotypical minicolumn core width was greatest in Brodmann 44 (BA44) (mean 16.5 μm; ±4.16 μm). The greatest difference between autistic and control groups was also observed in BA44, where autism related minicolumns averaged 10.3 μm (±2.69 μm). Since BA44 includes Broca’s area, the noted substantial loss of minicolumn architecture is likely a feature of the language impairment of autism. This area of the frontal cortex was also observed to be a source of abnormal magnetoencephalographic (MEG) patterns of epileptiform activity (Lewine et al., 1999). In addition, other observers find the superior temporal gyrus demonstrates significantly decreased functional connectivity, which was also accompanied by gray matter volume reductions (Mueller et al., 2013), thus reinforcing the indications for investigation of gray matter architecture.

**Figure 1:**
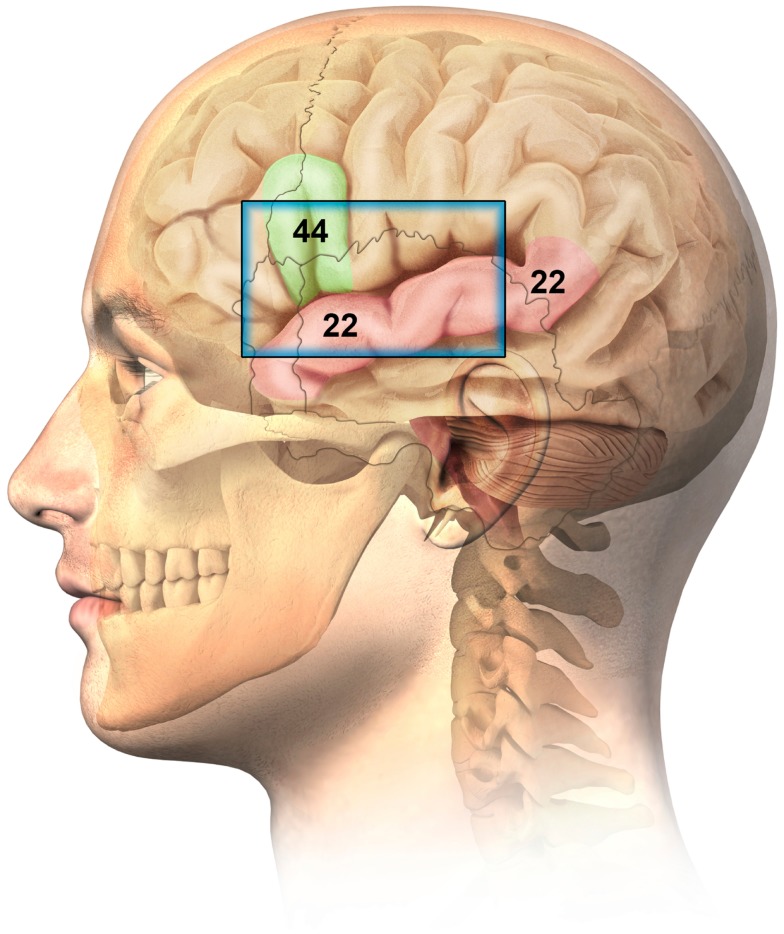
**Ultrasonographic window for the imaging of Brodmann 44 (Broca related language cortex) and Brodmann 22 (auditory cortex – superior temporal gyrus)**.

Anatomically, BA44 (part of Broca’s area) is situated beneath the left temporal-frontal cranium and is accessible via the squama temporalis bone (ST) and portions of surrounding anterior cranial boney structures. This represents an accepted and recognized window for TUS study of the brain, especially for children (American College of Radiology (ACR) (2012)), where the boney window allows a broader area of brain to be visualized. (Figure 1).

With adults, ST measurements determined that 56% represented ideal class 1 and 17% were satisfactory at class 2 (Jarquin-Valdivia et al., 2004). There was a noteworthy inverse relationship between ST thicknesses >5 mm and reduction in image quality. This was again confirmed when 19 of 37 (52%) adults proved to have adequate ST windows (Kollár et al., 2004). It is, however, naturally assumed children at younger ages provide a greater likelihood of successful ST ultrasonographic observations as is the case with sickle cell anemia and transcranial Doppler studies (Sarkar and Sharma, 2013).

The more posterior regions of BA22 are considered Wernicke’s area, which is classically noted to be involved in word recognition and processing (Dronkers et al., 2000), while the anterior portion of BA22 is considered primary auditory cortex. However, modern functional MRI imaging implicates broader areas of the STG than merely the posterior (Wernicke’s) portion of BA22 are involved in language processing (Harpaz et al., 2009).

Since BA44 and BA22 appear to be of both symptomatic, as well as histopathological significance in ASDs, and further are anatomically situated beneath an accessible TUS window, it was logical to evaluate these anatomies in ASD patients. Obviously, specific minicolumn architecture is below the resolution capacity of the best ultrasonographic technology, however, TUS resolution is potentially capable of observing structures as small as 0.1 mm. It was therefore hypothesized that TUS may allow for distinctions of anatomical significance in these critical cortical regions.

Further, MRI volumetric analyses of increased early neonatal extra-axial fluid (EAF) of siblings of children with autism, when not resolved by 24 months (up to three MRI scans required), was predictive of future autism development (Shen et al., 2013). While MRI is capable of greater specificity than TUS, its use as a screening tool, where three separate MRI procedures would be required, points to the need for a lower-cost screening technique. TUS has been used as a simple, economical, and reliable method of calculating EAF in the neonatal subarachnoid spaces (Lam et al., 2001; Libicher and Tröger, 1992). These processes employed standardized methodologies of ultrasonographic viewing of neonatal brain structures via the open frontal fontanel.

The primary purpose of this study was, therefore, to determine if the previously published observations of increased EAF persisted and could be assessed with TUS at later stages of development. TUS has also been recommended in the routine screening of low birth weight infants to determine the subsequent risk of autism (Movsas et al., 2013). So-called “benign” increased EAF is a common finding in survivors of neonatal intensive care units. The implications of the finding of increased EAF, however, call into question the benignity of the observation (Lorch et al., 2004). The aforementioned study used TUS in approximately half of the infants studied to measure the EAF. An observed increase in EAF, when compared to macrocephaly without increased EAF, predicted an increased risk of developmental delay and cerebral palsy.

## Materials and Methods

Transcranial ultrasonography is a U.S. Food and Drug Administration cleared procedure for children of all ages. The ethical considerations of TUS with both retrospective and prospective evaluations were further evaluated and approved by Liberty IRB, Inc., an independent investigational review board which is fully accredited. It is also registered with www.clinicaltrials.gov, registry number: NCT01980186. All families gave written permission for their child or children with ASDs, and where possible the subjects gave either consent or assent to diagnostic TUS. Participation was voluntary and services were provided at no cost to families. All procedures were performed as part of routine visits of the children or families whom were already existing patients.

### Normal TUS anatomy observed at the ST window

Our transcranial technique for viewing meningeal and cortical structures ultrasonographically has been previously published as part of a study of patients with myalgic encephalomyelitis and chronic fatigue syndrome (ME/CFS) (Pacini et al., 2012). The TUS normal anatomical observations were recently published by the *Italian Journal of Anatomy and Embryology* [volume 188(3) 2013, Figure 2].

**Figure 2:**
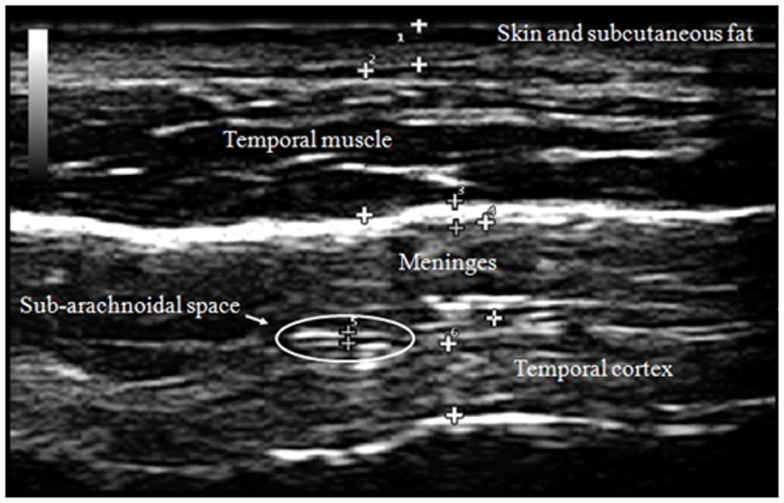
**Normal adult TUS anatomy in detail**.

**Figure 3:**
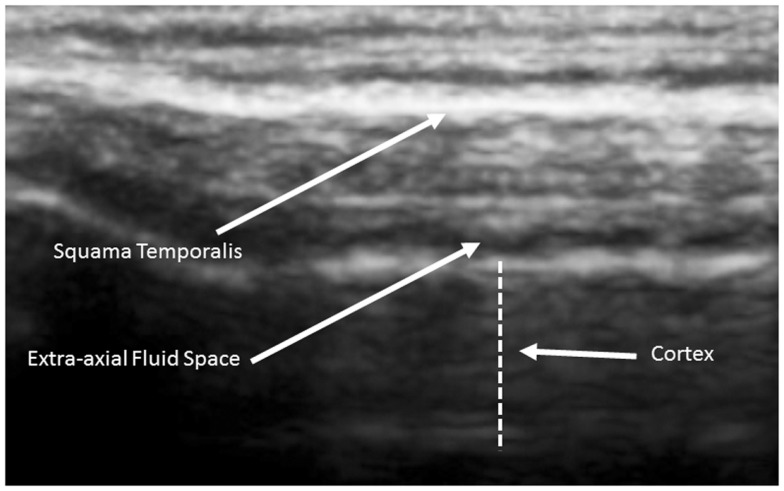
**Ideal view of the cortex from a 5-year old neurotypical sibling illustrating the full 4.5 cm width of the acoustical image**. Image is from SonoSite using non-magnified view.

The human cortex is known to vary between 2 and 4 mm (Kandel et al., 2000), and our observations are consistent with this. Generally, cortical thicknesses of BA44 and BA22 are 3.5–4.0 mm in children as old as 2 years and remains stable through young adult ages. The ideal TUS observations are of a portion of the cortex arranged parallel to the probe for at least 50% of the probe length (Figure 3).

**Figure 4:**
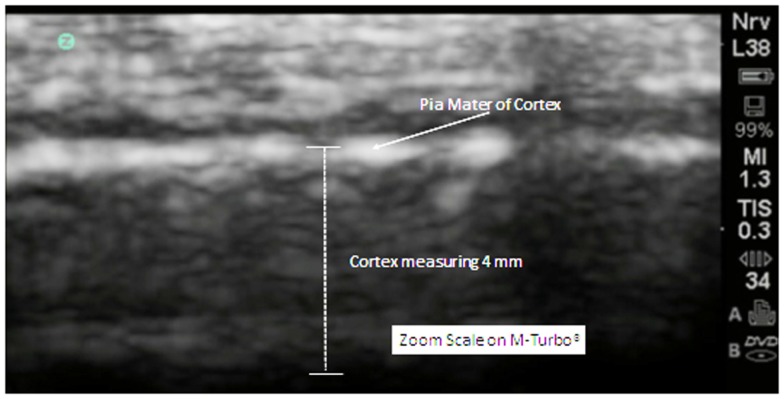
**In this zoom view from a 5-year old neurotypical sibling, the cortex can be observed to be uniform and layered in appropriate relationships**. There are no areas of hypoechogenicity within the substance of the cortex. The extra-axial space (subarachnoid) is modest but more than what will be observed at the gyral summit.

The cortex appears in linear and homogeneous layers representing the six distinct layers of the human cortex (Figure 4).

**Figure 5:**
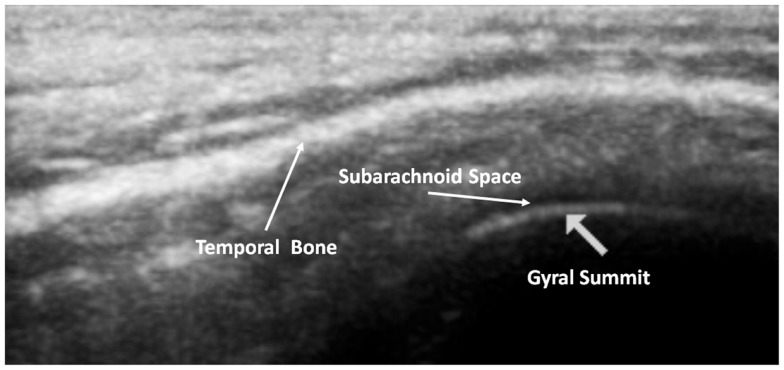
**Ideal image of 9 year old neurotypical sibling illustrating the normal gyral summit point for measurement of the EAF – subarachnoid space**. Here it can been seen to be minimal (at the lower limits of the calipers = 0.01 cm). Zoom mode image magnification from SonoSite.

The subarachnoid space is the interval between the arachnoid membrane and pia mater and it contains extra-axial cerebrospinal fluid. On the summit of each gyrus, the pia mater of the cortex and the arachnoid are in close proximity, and this is the area where qualitative estimates of EAF are best obtained (Figure 5).

**Figure 6:**
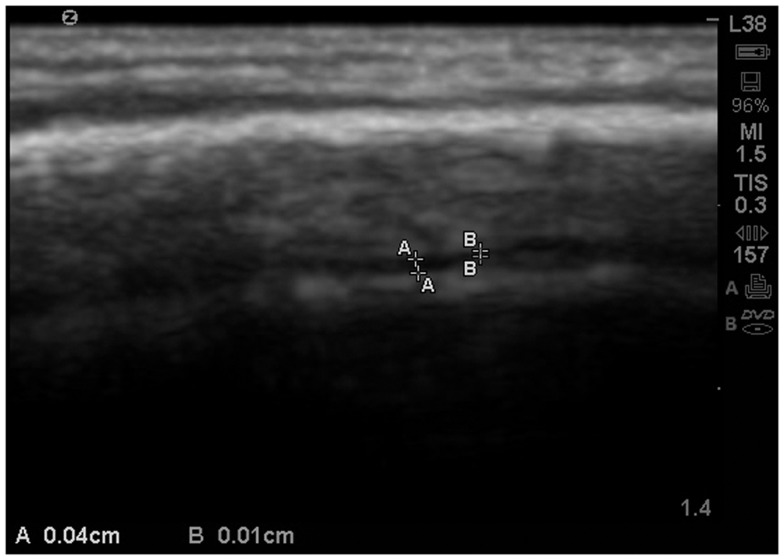
**Zoom view with SonoSite of the EAF varying between 0.04 and 0.01 cm at the summit**. At multiple locations the EAF space all but disappears. Sibling age 1.1 years.

As observed in that image, the EAF space is minimal, which is consistent with published observations of equally minimal EAF in children ages 2 and older (Lam et al., 2001), i.e., measuring <0.03 cm. The EAF is non-echoic and results in an enhancement of the echoes returning from the pia of the gyral summit, which often appears as a bright arched structure (Figure 5). Our observation in the healthy siblings studied indicate the arachnoid space at the gyral summit is <0.05 cm and often visible only as a fine dark line between the cortex and the arachnoid (Figure 6).

**Figure 7:**
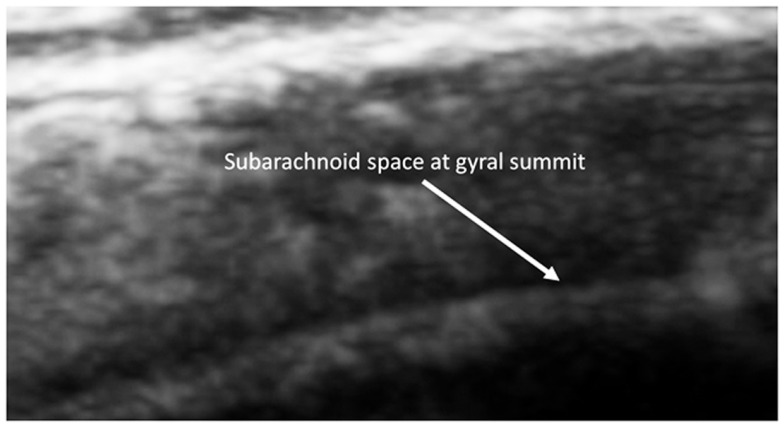
**Barely visible distance from the summit to the subarachnoid of a 2-year old sibling**. Magnified by the SonoSite zoom view.

This was also observed in neurotypical siblings of children with ASD at age 2, which is consistent with the observations of MRI scans from healthy siblings in the Shen et al., 2013 study (Figure 7).

### Procedure

**Figure 8:**
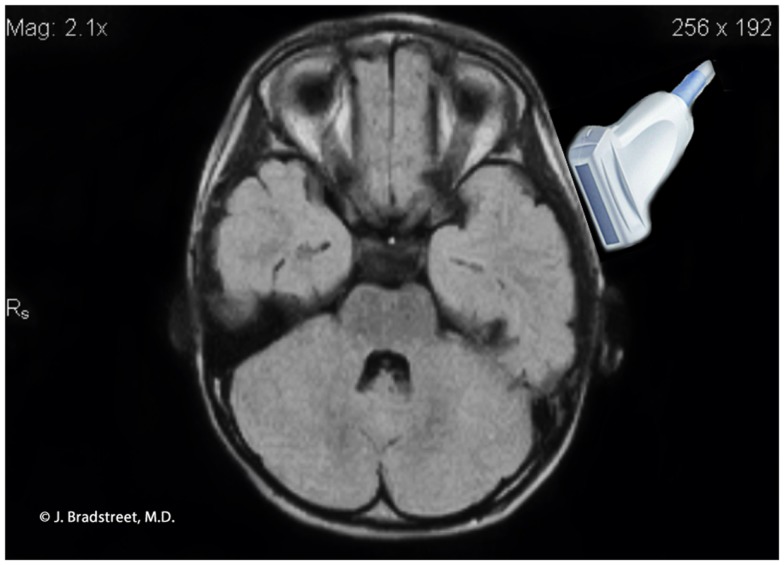
**MRI illustration of the anatomical relationship of the ultrasound probe to the auditory cortex**. The caudal lower limits of the probe’s ultrasonographic window.

For 2D TUS we used either an Esaote MyLabFive^®^ or the SonoSite M-Turbo^®^ ultrasound imaging machines approved for many applications including cephalic (brain) imaging. On the Esaote machine we used the default settings for adult transcranial imaging, but instead of a conventional transcranial probe, we used a linear probe (LA523) typically used for muscle-skeletal examinations, and we selected 7.5 MHz frequency. The majority of the cases and siblings were studied with the M-Turbo using the L38xi linear array probe (10 to 5 MHz), set with the preprogrammed “Nrv” protocol. The remainder were studied with the Esaote, using a power setting of 1.0. The acoustical aperture of each probe is approximately 4.5 cm. The cortex will vary by age, but the probe width and depth capabilities (9 cm for the L38xi) will include the necessary structures. The depth of the focus was varied according the localization of the anatomical structures to be studied and gain was set accordingly between 51 and 64% on the Esaote, or by adjusting the depth and gain on the M-Turbo^®^. To start, the probe is placed immediately anterior to left ear at about the level of the upper orbit and cephalad to the temporal mandibular joint (Figure 8). In all autism cases and siblings excellent to satisfactory image quality was obtained using this ST window.

It is important to use copious amounts of ultrasound gel to reduce any artifact generated by hair. The probe is then moved from the original position up the temporal window to explore both the temporal lobe and the Broca’s associated language cortical areas. Multiple images should be recorded and measurements of the EAF and the cortex need to be made during the process. A similar procedure is performed for the right temporal-frontal areas as well. The lower limit of measurement for the intrinsic software caliper of the SonoSite M-Turbo^®^ is 0.01 cm.

Several images were obtained and both the left (primary site of evaluation) and the right ST regions. On each machine and typically with all ultrasound hardware, the “freeze” setting stops the emission of the ultrasound. The technique employed used minimal exposure to the subjects by frequently “freezing” the image to take measurements and to evaluate findings.

### Image processing

The standard output choices for both the Esaote and SonoSite machines allow JPEG export of captured images. Typically, the images are captured from “frozen” recordings at both normal resolution and also “zoom” mode (a preset magnification process of the SonoSite software). Contemporaneous measurements of the EAF are recorded at the time of image capture. The JPEG images are then imported into Adobe^®^ Photoshop^®^ Essential version 11 for editing. Editing is generally limited to removal of the name of the patient and conversion to gray scale. Where necessary for print quality the brightness and contrast are adjusted to allow for improved print quality. No further editing is undertaken apart from the use of arrows or circles to identify the architecture of importance.

### Subjects

The study on autistic subjects was performed at the Brain Treatment Centers, Newport Beach, CA, USA and Buford (Atlanta), GA, USA. All procedures were performed by either authors James Jeffrey Bradstreet or Marco Ruggiero and reviewed by a certified clinical radiologist (Marco Ruggiero), whom while in the U.S. served as a sonographer under supervision of a licensed physician (James Jeffrey Bradstreet). With this initial cohort, parents volunteered ASD subjects (*N* = 23; males 18, females 5) for evaluations (all ages <16, mean = 7.46 years ± 3.97 years), and neurotypical siblings (*N* = 15; males 5, females 6) were also examined (all ages ≤16, mean = 7.15 years ± 4.49 years). No patients required separate sedation for the procedure, although two autism patients received standard pediatric doses of midazolam for phlebotomy procedures and had TUS performed following the oral sedative. No sibling reported any symptoms related to TUS investigations.

As far as autistic subjects are concerned, all records were reviewed by James Jeffrey Bradstreet for confirmed diagnosis of autism and the observed clinical/parental/therapist/teacher ratings. All autism subjects met the Diagnostic and Statistical Manual of Mental Disorders (DSM-IV-TR^®^), 299.00 criteria, and were separately diagnosed by either a child neurologist or developmental pediatrician in addition to receiving the evaluation of the experienced clinician (James Jeffrey Bradstreet). In the routine course of evaluation, all autistic subjects were rated using the Childhood Autism Rating Scale-2 (CARS2^®^) (Schopler et al., 2010).

We further created a scoring system for both cortical dysplasia and EAF using the following criteria. Here we define cortical dysplasia as hypoechoic lesions within the substance of the cortex, or disturbed layering within the gray matter. This new system for cortical dysplasia and EAF scoring is presented in Table 1.

**Table 1:** **A system of scoring cortical dysplasia and EAF**.

Grade	Cortical dysplasia classification
1	None observed
2	Rare hypoechogenic lesions and/or mildly atypical cortical layering patterns
3	More common, but separated areas of cortical dysplastic lesions, or more commonly abnormal cortical layering
4	Very common or confluent areas of cortical dysplasia and/or markedly disturbed layering of the cortex

**Grade**	**Extra-axial fluid classification**

1	<0.05 cm
2	0.05–0.07 cm
3	0.08–0.10 cm
4	>0.10 cm

*Examples of varying degrees of cortical dysplasias will be presented in the results section*.

## Results

Of the neurotypical siblings measured, none presented with issues related to increased EAF or CD: whereas all but one of the autism subjects (22 of 23) had evidence of grade 2 or greater cortical dysplasia (mean = 2.79 ± 0.93) apparent at the ST window related cortex (BA44 and 22) (Table 2). For EAF, all 23 autistic subjects demonstrated increased EAF at the gyral summits when compared to neurotypical siblings (mean EAF score 3.41 ± 0.67). Further, 91.3% representing all but 2 of 23, had an EAF judged to be 3 or 4; of which 13 of 23 (56.5%) presented with EAF >0.10 cm. In further analysis of this subset (EAF >0.10 cm), we observed a mean CARS2^®^ score of 50.5 ± 6.5.

**Table 2:** **Results of TUS studies in the ASD group compared to their neurotypical siblings**.

									
Siblings		Age	ASD cases		Age	CARS	CARS XV	Cortical dysplasia	Extra-axial fluid
								Score	Score
S1	M	5.5	A1	M	12.4	52	4	3	3
S2	M	6.2	A2	M	5.6	54	4	3	3
S3	M	16.4	A3	M	8.8	44	3.5	1	3
S4	M	13.4	A4	M	15.3	57.5	4	4	4
S5	F	12	A5	M	10.5	55	4	4	4
S6	F	8.1	A6	F	3.8	53.5	4	4	4
S7	M	7.9	A7	M	15.6	55	4	4	4
S8	M	6.5	A8	M	12.8	56.5	4	4	4
S9	F	2.1	A9	M	4.3	49	4	3	3
S10	F	9.1	A10	M	3.8	37	2.5	2	2
S11	M	2.7	A11	M	3.1	49	3.5	3	3
S12	F	10.9	A12	M	5.9	44	3.5	3	3
S13	M	4.7	A13	M	8.7	51.5	4	4	4
S14	M	4.2	A14	M	5.8	48	3.5	3	3
S15	M	10.3	A15	M	7.7	57	4	4	4
	Mean	7.15	A16	M	7.8	34.5	2.5	2	2
	SD	4.49	A17	M	10.9	52.5	4	4	4
			A18	M	4.7	40.5	3.5	2	4
			A19	M	15.8	36	3	2	4
			A20	F	6.11	49.5	4	2	4
			A21	F	9.1	44	3	2	3
			A22	F	9.3	50.5	4	2	4
			A23	F	4.2	46.5	4	3	4
				Mean	7.46	48.08	3.64	2.79	3.41
				SD	3.97	6.79	0.49	0.93	0.67

*The scores related to EAF and cortical dysplasia are based on the rating system in Table 1. CARS2^®^ scores reflect the rating at the time of testing. CARS2^®^ question XV is the global impression of autistic severity from the CARS2^®^ questionnaire. Mean (geometric mean) and SD are calculations from Microsoft Excel^®^ 2013 formulae*.

As noted in the table, we assessed both the total CARS2^®^ score and the score for question XV, the general impression score for autistic severity. The CARS2^®^ scores were obtained as a routine in the practice and the ASD score mean was 48.08 ± 6.79 (range 34.5–57.5). All but one of which were defined within the severe range of the metric, and only 3 scored below 40.

**Figure 9:**
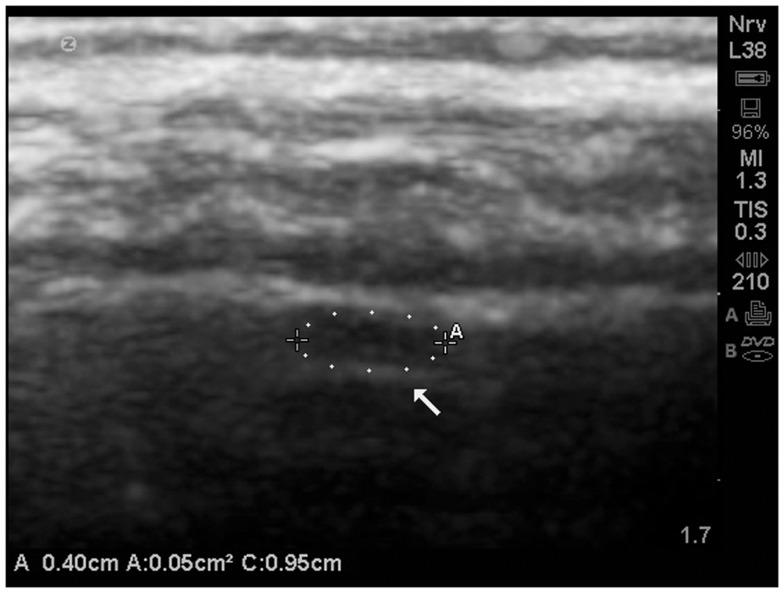
**Isolate cortical lesion compatible with a grade 3 lesion due to its size**. SonoSite zoom magnification. The arrow points to the enhancement distal to the hypoechoic area which indicates this is unlikely to be an artifact.

An example of an isolated grade 3 lesion is noted in (Figure 9). It is located in the subpial region and appears as a decrease in echogenicity with a distal to the probe enhancement artifact.

**Figure 10:**
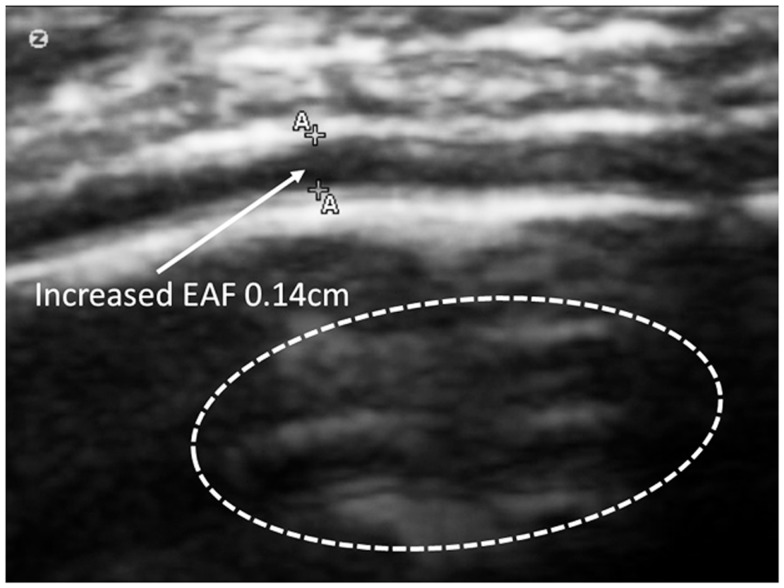
**Zoom mode of gyral summit of 12-year old ASD subject with significant enlargement of the EFA – subarachnoid space**. This view also demonstrates multiple areas of cortical dysplasia (circled) grade 4. SonoSite zoom mode.

In several cases, these cortical dysplastic lesions were common (grade 4), and often multiple lesion were demonstrated on the same view (Figure 10).

**Figure 11:**
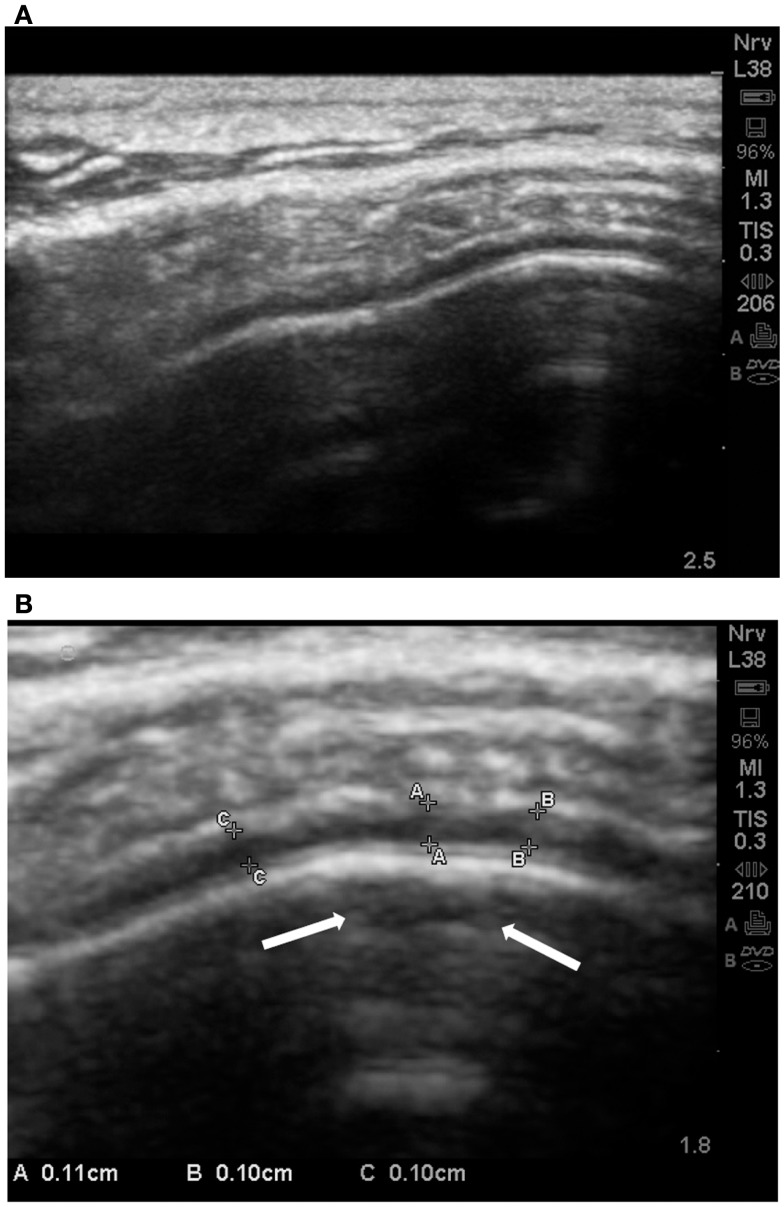
**(A) Standard SonoSite capture without magnification of the gyral summit of a subject with autism**. The compression of the arachnoid volume by the gyral summit is apparent. **(B)** This is the same child and the same gyral summit location as 11a. It is the out-processing of the zoom software of the SonoSite. Multiple measurements are taken between the pia and the subarachnoid membrane. All are between 0.10 and 0.11 cm. In the subpial region of the summit there is an area of hypoechoic reflection with distal enhancement (arrows); grade 2 CD.

In 8 of 22 ASDs cortical dysplasia was rated as 4 (severe). Cortical dysplasias were always observed in association with increases of the subarachnoid space [≥0.05 cm at the gyral summit; grade 2–4 (mean = 3.41 ± 0.67)]. We noted that of the autistic subjects, 21 of 23, revealed increases in the subarachnoid spaces at grade 3–4 and sometimes cortical dysplasia was noted at the summit (Figures 11A,B).

**Figure 12:**
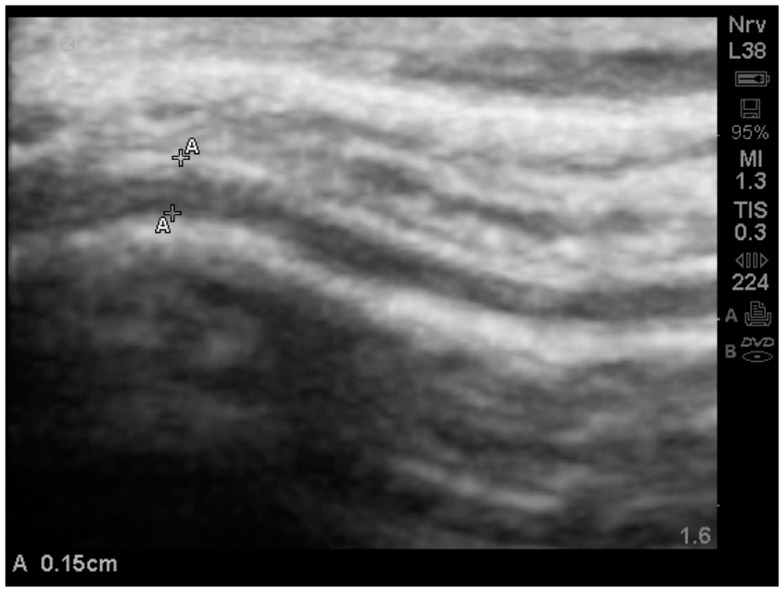
**Substantially increased EAF from the left frontal cortex – subarachnoid space (*A*–*A* = 0.15 cm) grade 4**. Image from subject A19. SonoSite set at zoom mode.

Often times the increased EAF space was dramatic; >0.1 cm as noted in (Figure 12).

**Figure 13:**
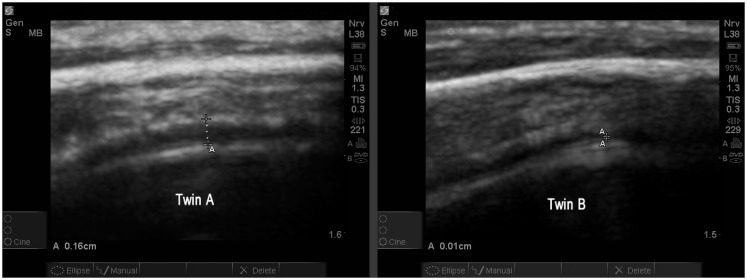
**Twin A with autism demonstrates a significantly increased EAF (in the range of 0.11–0.16 cm measured at various locations) (SonoSite zoom view)**. In contrast with his autistic brother, neurotypical Twin B has an obvious narrowing of the EAF at the gyral summit (0.01 cm) SonoSite zoom view. Twin A appears to have a somewhat thicker temporal bone; despite this the image quality is very good. Both images show anatomically similar areas of BA44.

The study allowed one direct comparison between fraternal twins. The boys, age 4.7 years, are compared for EAF and CD. Twin A with autism (Figure 13) is noted as A18 (Table 2) and his unaffected sibling (Figure 13, Twin B) is subject S13 in the same table. The images are from a similar cortical area (BA44) and are both represented as SonoSite Zoom resolutions.

**Figure 14:**
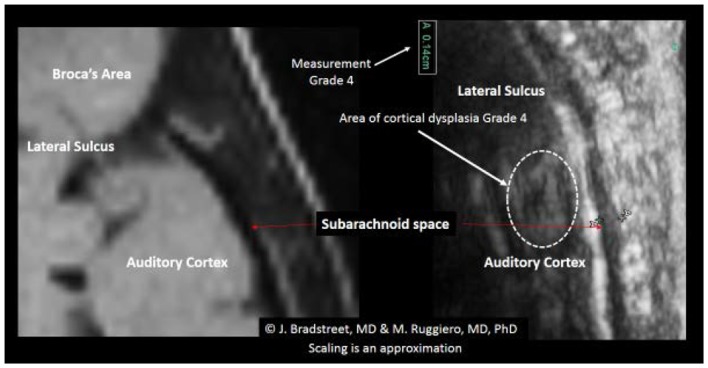
**The side-by-side comparison illustrates the differences in imaging obtained by MRI and TUS**. The ultrasound image was rotated 90° to align with the MRI. SonoSite at regular image size (not zoom). In each the EAF is observable as increased, but typically radiologist do not attempt to quantify the EAF from MRI. Here the TUS measurement of EAF is 0.14 cm (grade 4). The circle illustrates an area of apparent cortical dysplasia which resembles the architecture of the cortical dysplasias reported by Wegiel et al. (2010).

When available, the study protocol allows us to compare MRI to TUS. While we anticipate subsequently publishing further observations on this process (Figure 14), presents a side-by-side comparison of one subject’s (A15, Table 2) MRI and TUS studies. The MRI was obtained overseas, while his mother and he were visiting family. The MRI is from a 0.5 T machine and was interpreted as normal by a local radiologist. While 0.5 T MRI technology is considered subpar by most advanced radiologist, the image at least allows for localization and comparison of macro-anatomical features.

**CHART 1:**
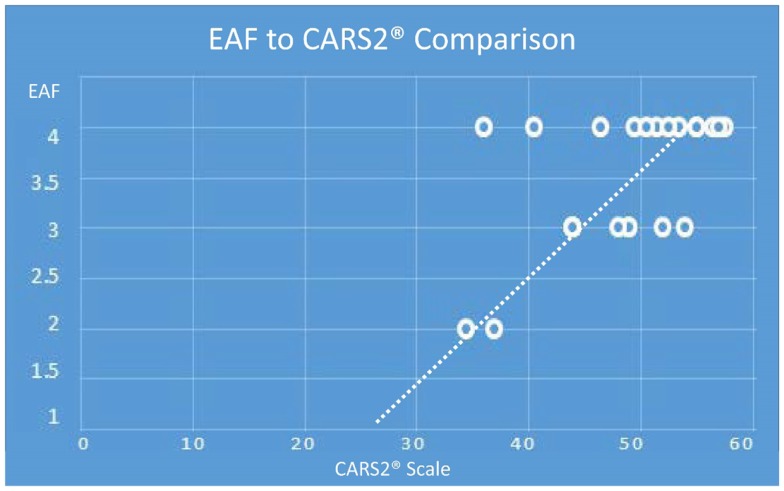
**(Note: the maximum EAF score is 4.0, minimum is 1.0 and the maximum CARS2^®^ is 60 and the minimum score is 15)**. Depending on age, the cutoff for an autism spectrum rating by CARS2^®^ is 27–30. Extrapolating the data illustrates an EAF score of 1, is associated with a non-autism CARS2^®^.

There was also a general trend to see more cortical lesions on the left hemisphere than on the right. However, in cases where the children were more severely autistic, cortical lesion were common in both hemispheres. As noted in Table 2, there were apparent trends for increased severity scores on either the total CARS2^®^ or the general severity score (question XV), to be associated with higher cortical and EAF scores. With the one autistic subject where no cortical lesions were identified, we did observe significant increased subarachnoid spaces (grade 3). Although the sample size was relatively small, Chart 1 illustrates the relationship of CARS2^®^ to EAF and there is an apparent trend toward higher EAF being associated with more severity on the CARS2^®^ scale.

**CHART 2:**
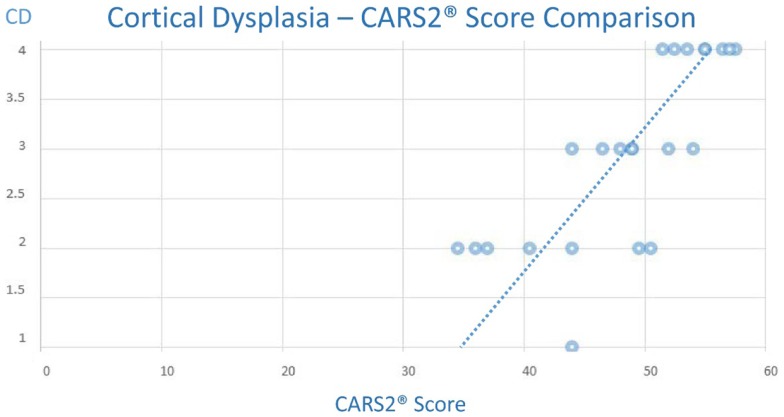
**(Note: the maximum CD score is 4.0, minimum is 1.0 and the maximum CARS2^®^ is 60 and the minimum score is 15)**.

Equally, we noted a trend of increased CARS2^®^ was also associated with an increased CD score (Chart 2), although the trend appeared less predictive of an autism associated CARS2^®^ score threshold.

None of the neurotypical siblings, regardless of age, presented with any cortical dysplasias as were noted in the autistic subjects. Neither did they demonstrate increased subarachnoid spaces at the gyral summits.

Of special note, one subject (A23), was noted to have a mass lesion within the meninges (Figure 14).

**Figure 15:**
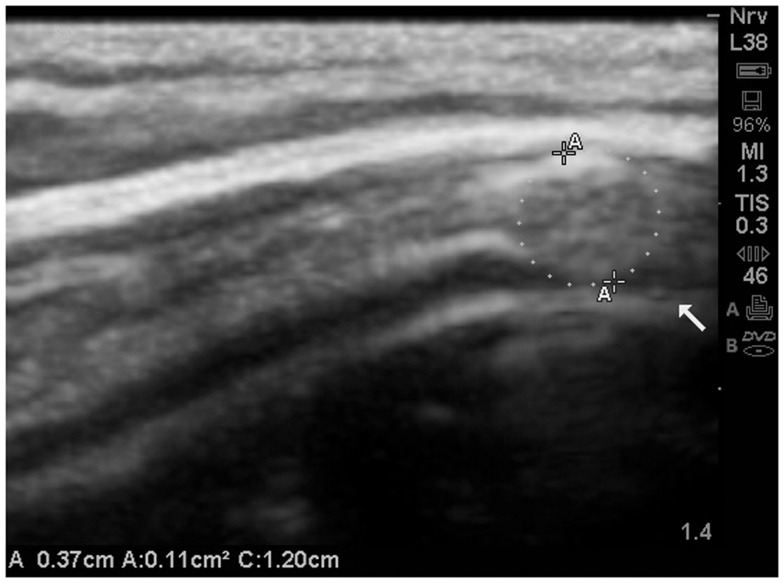
**The subarachnoid space at the gyral summit demonstrates abrupt closure secondary to a mass effect within the meninges**. Further, the gyral summit can be observed to flatten somewhat by the effect of meningeal mass. This is a presumed meningioma and most likely benign. The patient has been referred for MRI and further consultation. MRI is not presently available.

The image (Figure 15) is presented merely in the context of the ability to visualize the subarachnoid space at the gyral summit, and in this case the closure of the space is clearly evident on the ultrasound image.

## Discussion

To our knowledge, these observations represent the first ultrasonographic investigations of the autistic central nervous system condition. The precise nature of the hypoechoic cortical lesions observed is uncertain. They would seem to be pseudocystic based on their distinctive appearances in some cases. While these could represent artifacts, both the nature of the reflectivity and the absence of similar hypoechoic cortical findings in neurotypical siblings, argue against these images representing artifacts. More likely, these lesions represent focal cerebral edema or perhaps areas of poor cell connectivity and hence limited reflectivity. The dimensions of the lesions are generally below the resolution of routine MRI studies of children (in the U.S. most scanners used in pediatrics are 1.0–1.5 T) where investigations utilize 5–6 mm slices (gaps). However, newer MRI machines with 3.0–7.0 T capacity could easily image the lesions we observed if detailed studies at 1 mm or less were utilized. Given both the cost and the relative scarcity of ultrahigh resolution MRI technology, we propose, that if the observations of this limited initial study are replicated, that TUS methods of screening be used prior to referral for more complex imaging.

We also suggest that these areas of apparent cortical dysplasias, if confirmed by high resolution MRI, may present a new, real-time observation of diagnostic significance. Due to the individual circumstances regarding the causes of death related to brain-banked specimens, the cortical lesions may be less obvious when post-mortem, formalin-fixed sections are evaluated. Nonetheless, if confirmed by high-resolution MRI, the etiology of these lesions warrants further detailed examination to determine whether active inflammatory disease is present, or whether these findings represent developmentally disordered cellular distribution patterns. It is further possible these are clusters of abnormal minicolumns. Using the previously cited metrics (Casanova et al., 2002, 2010), it would seem 100 to >500 minicolumns may be represented per lesion.

The increased EAF estimates we observed to be persistent in all subjects with autism are consistent with the previous mentioned MRI investigation by (Shen et al., 2013) of infant siblings of children with autism whom themselves went on to develop autism. The lack of resolution of increased EAF by age 2 was predictive of a subsequent autism diagnosis. Our estimated increased EAF assessments are also consistent with the previously mentioned findings related to NICU survivor risk of developmental delays when EAF was observed elevated at early ages. Crucially, if one examines the MRI presentations of (Shen et al., 2013) closely, it is apparent the gyral summits of the children not destined to develop autism come in close contact to the subarachnoid membranes. This is an observation we observed via TUS in our neurotypical sibling population. Of equal importance, the MRI images of (Shen et al., 2013) illustrate significant gaps between the gyral summit and the subarachnoid membranes of the children whom eventually develop autism. This is precisely what the TUS observations of this study illustrated.

Abnormally increased EAF has potential links to inflammatory changes secondary to stagnation of the cerebral spinal fluid (CSF) flow and the prolonged exposure to toxic byproducts of metabolism and chemical messengers of inflammation (Johanson et al., 2008). It further appears the quality of the CSF is key factor in neuronal development within the cortex (Miyan et al., 2006). As with the discussions of both (Shen et al., 2013) and (Lorch et al., 2004) the nature of the why increased EAF is an indicator of subsequent risk of developmental delays is speculative, but warrants more detailed investigations.

The observations of this study, taken with the observations of (Shen et al., 2013), point to a possible role of the meninges in the pathophysiology of autism. Once thought to be of little significance to cortical pathology, newer observations indicate the meninges participate in migraine (Levy, 2009), and potential contribute to multiple sclerosis (Gardner et al., 2013). The Gardner group from Imperial College in London hypothesized that pro-inflammatory cytokine production within the meninges may be a key to the demyelination model they created for multiple sclerosis. Mast cells, which are prevalent in both the meninges and regions of the cortex, have been hypothesized to participate in autism pathophysiology via disruption of the blood-brain barrier (Theoharides and Zhang, 2011).

The volume of fluid in the EAF, like all extracellular fluid spaces, is the consequence of complex interstitial fluid mechanics (Yao et al., 2012). Thus the increase in endovascular permeability suggested as a consequence of mast cell activation in autism by Theoharides and Zhang, would consequently result in an alteration of the fluid dynamics in the EAF. Presumably, the pressure relationship between the end-capillary pressure, resultant increased extravasation of fluid, and the hydrostatic pressure of the subarachnoid reach a new equilibrium which results in increased EAF.

Immunological investigations from ASD spinal fluid assessments has yielded inconsistent observations. Messahel et al. (1998), observed CSF levels of neopterin (a marker of monocyte activation) to be approximately three times that of neurotypical controls. In contrast (Zimmerman et al., 2005), observed what they felt were paradoxically lower levels of neopterin occurring with only elevated tumor necrosis factor (TNF) receptor II elevations above that of controls. In a separate study (Chez et al., 2007), compared simultaneously acquired serum and CSF levels of TNF-alpha and found dramatically higher levels of TNF-alpha in the CSF compared to the serum of autistic subjects. These observations point to the importance of the EAF assessments in ASD individuals.

### Safety observations

Recently, the safety of prenatal ultrasonography was evaluated in a large prospective study of over 1,000 pregnancies in Australia; no increased risk of autism was noted after five ultrasonographic evaluations during the course of the pregnancies (Stoch et al., 2012). Further and as noted by Hameroff et al. (2013), TUS is considered safe and its diagnostic application is approved at these frequencies without consideration for time of exposure. One subject in that study experienced transient exacerbation of headache symptoms which resolved quickly without any complications noted up to 4 months post-TUS. In our observations, none of the siblings reported any side-effects. Adding further reassurance to the safety factor of TUS is the study of Shimizu et al. (2012). In that study, they applied mid-frequency TUS (0.1–1 MHz) ultrasonic thrombolysis for acute ischemic stroke to monkeys and included diagnostic 2D mode studies at 2.5 MHz. Despite far greater exposure and acoustical power with an associated increased risk of acoustical cavitation from that procedure, none was observed. As such, TUS properly administered at the frequencies and times of exposures in this study are considered safe.

### Limitations of this study

While TUS is able to look with high resolution at specific areas of the cortex valuable to the assessment of ASD, it has limitations. The cerebellar vermis and other posterior fossa anatomical structures of significance to autism (Bolduc et al., 2012) have yet to be observed with TUS in autistic subjects. Potentially, the foramen magnum could provide another ultrasonographic window for investigation of these structures (Brennan and Taylor, 2010), but as of this writing, methodologies applicable to autism remain uncertain. A further limitation of TUS is the inability to measure basal ganglion and thalami (Estes et al., 2011), as well as the amygdala (Bellani et al., 2013); all of which are centers of interest to ASD research. Neither can our TUS estimates provide EAF volumetric analyses. These measurements do, however, indicate qualitative observations of increases in the subarachnoid space at the gyral summit which appear consistent with other observations of abnormal EAF in ASD.

Asymmetry within the language associated cortex has been reported with detailed MRI volumetric analyses in both autism and specific language impairment (SLI) (Herbert et al., 2002; De Fossé et al., 2004). It appears to correlate with the degree of language impairment rather than autism *per se*, since it was observed in both SLI and autism. In the autism population, the asymmetry was correlated with the degree of language impairment. It is doubtful, however, that TUS could be used to assess these changes. Our early and unconfirmed TUS observations of possible asymmetry between the right and left cortices are not related to volume measurements, but rather to observations of focal dysplasia. The observations of the cortical differences was, initially, an unanticipated finding. This present study, while able to image at adequate depth, was not designed to measure cortical thickness in the autism population.

The study populations consist of individuals with autism and their neurotypical siblings. This study was not designed to find unrelated controls via an age and gender matched case-controlled methodology. It was merely hoped we could contrast healthy siblings and individuals with autism for differences in the EAF and possibly observe cortical dysplasias within the autism population. Of particular interest is the direct comparison of a set of fraternal twins (only one with autism) in our study. The differences of EAF between the two brothers is obvious and compelling.

We recognize a challenge to any ultrasonography measurement of the dimensions of the brain and its EAF spaces is the convolutions of the gyri compared to the angle of the probe. Oblique angulation of the probe could exaggerate the measurement of thickness. While we attempted to reduce this variable with multiple measurements and varying of the probe angle, this is still an operator dependent procedure, and thus subjectively based on the observer’s choices of images to capture.

## Conclusion

Despite the various limitation of TUS, this study represents a simple, reproducible, safe and effective, yet low-cost methodology to examine cortical areas of significance to autism (BA44 and BA22) along with qualitative assessment of the EAF volume. Given the negative impact of autism for quality of life and the lifelong expenses for families and society, TUS could be implemented as a screening tool in the neonatal period, and at-risk children whom were determined to have increased EAF or cortical dysplasia, could be referred for careful monitoring and perhaps more detailed examination with MRI. Whether early intervention would prove therapeutic is a subject for future studies, however the implications for therapeutic clinical research appear significant, since these are real-time observations of the living subject.

## Conflict of Interest Statement

No physician associated with this study received any compensation for the study, writing, or examinations involved with these observations. James Jeffrey Bradstreet is a practicing clinician in the U.S. who uses ultrasonography in his practice of medicine. James Jeffrey Bradstreet is also the father of a child with autism (not a participant in this study). Marco Ruggiero is a certified radiologist in Italy with an academic appointment with the University of Firenze. Stefania Pacini has no conflicts of interest.
